# Fad Diets: Facts and Fiction

**DOI:** 10.3389/fnut.2022.960922

**Published:** 2022-07-05

**Authors:** Aaiza Tahreem, Allah Rakha, Roshina Rabail, Aqsa Nazir, Claudia Terezia Socol, Cristina Maria Maerescu, Rana Muhammad Aadil

**Affiliations:** ^1^National Institute of Food Science and Technology, University of Agriculture, Faisalabad, Pakistan; ^2^Department of Genetics, University of Oradea, Oradea, Romania

**Keywords:** fad diets, obesity, weight loss, metabolism, chronic disease, cardiovascular, health

## Abstract

The global prevalence of obesity is alarmingly high and is impacting both developed and underdeveloped countries, beyond the borders of ethnicity, sex, and age. On the other hand, the global interest in dieting has increased, and people are obsessed with certain fad diets, assuming them as a magic bullet for their long-term problems. A fad diet is a popular dietary pattern known to be a quick fix for obesity. These diets are quite appealing due to the proposed claims, but the lack of scientific evidence is a big question mark. Such diets are often marketed with specific claims that defy the basic principles of biochemistry and nutritional adequacy. These diets may have protective effects against obesity and certain chronic diseases like cardiovascular diseases, metabolic syndrome, and certain cancers. Limited evidence exists to support the proposed claims; rather certain studies suggest the negative health consequences of long-term adherence to such dietary patterns. Many fad diets have emerged in the previous few decades. This review article will explore the current evidence related to the health impacts of some most popular diets: Atkins diet, ketogenic diet, Paleolithic diet, Mediterranean diet, vegetarian diet, intermittent fasting and detox diet.

## Introduction

Obesity is one of the major public health concerns in this modern era. It is now considered a global epidemic due to the gradual but continuous increase in its prevalence. The global prevalence of obesity is alarmingly high and is impacting both developed and underdeveloped countries, beyond the borders of ethnicity, sex, and age. Worldwide obesity has tripled from 1975 to 2016, while childhood obesity is increasing dramatically (1). Excessive calories from fats and sugars, large portions of food, routinely junk food intake, availability of fast foods at the doorstep and limited physical activity are some of the contributing factors to obesity (2). Obesity is an independent risk factor for morbidity and mortality. Being obese or overweight puts a person at greater risk of developing cardiovascular diseases, hypertension, insulin resistance, diabetes, reproductive issues, liver and kidney diseases (3).

**FIGURE 1:**
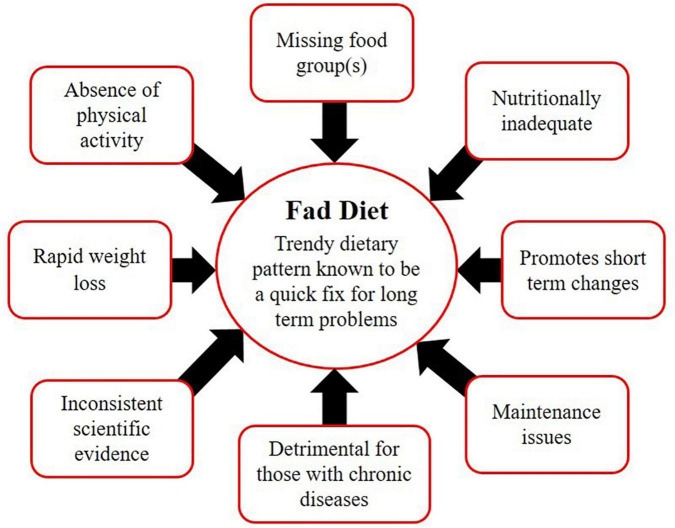
Characteristics of fad diets.

Despite the growing global prevalence of obesity, there is always a group that is highly obsessed with dieting. The global interest in dieting has increased in the last two decades. A study indicated that internet searches related to weight loss queries had immensely increased between the years 2004 to 2018 (4). In the meantime, people rush toward certain fad diets (FD), assuming them as a magic bullet for their long-term problems. FD is not a scientific terminology but rather a popular or trendy dietary pattern that is known to be a quick fix for obesity (5). FD can be easily differentiated from a healthy and balanced diet based on its characteristic features: (i) promises rapid weight loss (ii) absence of physical activity guidelines (iii) promotes short-term changes rather than achieving lifelong sustainable goals (iv) focuses on one type of food or eliminates any food group (v) cannot be maintained for life long period (vi) nutritional adequacy is questionable (vii) fails to provide health warnings for those with chronic diseases (viii) lacks scientific evidence to support the claims (5, 6) (Figure 1).

## Aim of the Study

A wide range of FDs has been proposed to date, ranging from low carbohydrate diets to low-fat diets, high-fats to high-protein diets, those with detoxification claims, and others of the Mediterranean or Paleolithic origin. These diets are followed blindly but are associated with certain negative health outcomes as one size does not fit all. This review article will explore the current evidence related to the health impacts of some popular diets, including Atkins diet, ketogenic diet, Paleolithic diet, Mediterranean diet, vegetarian diet, intermittent fasting, and detox diet.

## Atkins Diet (AD)

In the 1970s, a low carbohydrate, high protein (LCHP) regimen was developed by cardiologist Dr. Robert Atkins, which was published in his book “Dr. Atkins’ New Diet Revolution” (7). This diet was promoted as a quick weight loss plan based on a lifetime change in eating habits. Atkins believed that metabolic imbalance resulting from carbohydrate consumption is the major cause of obesity. He claimed that this is the easiest, high-energy diet that mobilizes fats more than any other diet for weight loss maintenance. The AD involves an extreme reduction of carbohydrates, i.e., less than 5% of total calorie intake, *ad libitum* intake of proteins and fats, adequate fluid intake with vitamin and mineral supplementation, and regular exercise (8).

The diet has four phases: induction phase, ongoing weight loss phase, pre-maintenance phase, and lifetime maintenance phase (Table 1). The modified version of the AD (MAD) is currently available with the same four phases but slightly modified net carbs consumption in each phase. The MAD is less restrictive, allowing the person to choose the number of net carbs in phase 1, i.e., 20, 40, or 100 g of carbs and fats are not just allowed but encouraged. The primary goal is not weight loss rather it has shown promising results in seizure reduction in intractable epilepsy (9–13).

**TABLE 1:** Phases of the Atkins diet.

Phases	Duration	Major considerations	Food sources allowed	Reference
Phase 1: Induction	2 weeks	Carbs restriction to <20 g/day	Protein-rich foods: beef, poultry, fish, egg, etc., good fats: olive oil, etc.	(8)
Phase 2: Ongoing weight loss	Variable (until weight loss cease)	Gradual increase in carbs intake at a rate of 5 g per week	Nutrient dense carbs, proteins and fats	
Phase 3: Pre-maintenance	Variable (Addition phase till weight loss continues, cut back until weight loss resumes)	Additional 10 g carbs per week Cut back 5 to 10 g carbs when weight loss resumes	Nutrient dense carbs, proteins and fats	
Phase 4: Lifetime maintenance	Lifetime	Addition of a wide range of foods while keeping carbs in check, i.e., 40–90 g net carbs a day	Nutrient-dense carbs, proteins and fats	

### Effectiveness of Atkins Diet

There is substantial evidence suggesting that AD promotes more weight loss than conventional diets. One of the first AD research was published in The New England Journal of Medicine in 2003. Brehm et al. (14) in a study allocated 53 healthy, obese women to two groups, i.e., low carbohydrate ketogenic diet (LCKD) or energy-restricted low-fat diet (LFD) (carbs: 55%, protein: 15%, fats: 30%). Over 6 months, the LCKD subjects lost 8.5 kg versus 4.2 kg in the LFD group. There were no comparable differences between the groups in serum glucose, lipids, leptin, and insulin excluding triglycerides that showed a significant reduction in the LCKD group.

In another randomized trial, 132 severely obese individuals (43% had metabolic syndrome while 39% had type 2 diabetes) were assigned to two groups. One group followed AD and the other followed LFD for 6 months. The results showed that LCD individuals lost 3.8 kg more weight than those on LFD. No significant difference was observed in both groups after 12 months (15). In another controlled trial of 1 year, 63 obese participants were randomly assigned to either the AD or conventional LFD. After 6 months, results showed that the LFD group lost less weight, i.e., 3.2 ± 5.6% than the AD group, i.e., 7.0 ± 6.5%. The AD group lost 4% more weight, had higher levels of high-density lipoprotein cholesterol (HDL-c) and lower levels of triglycerides (TG) than the other group. No significant differences between groups were noted in low-density lipoprotein cholesterol LDL-c (16).

Several meta-analyses and systemic reviews reported the promising effects of low carbohydrate diets on weight loss and cardiometabolic risk factors. Mansoor et al. (17) demonstrated that the LCD group had a significant increase in HDL-c and LDL-c, and had a greater weight loss and TG reduction in contrast to those following LFD. Hashimoto et al. (18) reported that LCD resulted in a greater reduction of body weight and body fat mass than the control diet. LCD was linked with moderately more significant advancement in weight loss and reduction of atherosclerotic cardiovascular diseases (ASCVD) risk, compared to LFD (19).

Naude et al. (20) concluded that both LCD and balanced diets had shown weight loss. After 2 years of follow-up, there was no significant difference between the diets in terms of cardiovascular and diabetes risk factors. Bueno et al. (21) found that after 12 months or more, the individuals that followed an energy-restricted very low carbohydrate diet (VLCD) (carbs: <50 g/day or 10%) compared to LFD (fats: <30%) had a more significant improvement in HDL-c, LDL-c, TG and diastolic blood pressure (DBP) as well as the reduction in body weight. Hu et al. (22) compared LCD and LFD and concluded that both diets were efficient at reducing waist circumference, body weight, total cholesterol (TC), total to HDL-c ratio, LDL-c, TG, blood glucose, serum insulin, and blood pressure. LCD showed a greater decrease in TG, and less reduction in LDL-c and TC but increased HDL-c in comparison with LFD.

### Health Consequences

Atkins diet has not been extensively studied while those studies that have been mentioned earlier have high dropout rates and are sometimes non-conclusive. Despite the rapid weight reduction, there are some concerns for those with comorbidities. There are some considerable potential complications associated with LCHP diets. There is conflicting evidence on the urinary stone formation tendency of LCHP diets (23). A short-term study showed that healthy subjects followed the LCHP diet for 6 weeks, decreased urine pH, increased urinary-acid excretion, and decreased calcium balance was observed in them. Therefore, they had a greater risk of stone formation (24). A prospective cohort study was conducted in Iran, involving 1,797 participants that were followed up for almost 6 years. Results showed that a higher tertile of LCHP diet correlates with a greater risk of chronic kidney disease (CKD) (25).

Metabolic acidosis is a common complication of LCHP diets. A case of 40 years old obese woman was reported, who was presented with nausea, vomiting, dehydration, and dyspnea. Investigations revealed that she was following AD, lost 9 kg in 1 month, and laboratory findings were consistent with ketoacidosis Chen et al. (26). Pregnant and lactating mothers should be cautious when following such a diet as there is a reported case of LCD-associated ketoacidosis in a non-diabetic lactating mother (27). AD provides several benefits including weight reduction and cardio-metabolic health improvement, but limited evidence exists as compliance is the major barrier to this dietary regimen. Strict supervision by health professionals is advised as adverse metabolic sequelae can result from this type of diet.

## Ketogenic Diet (KD)

In 1923, Dr. Russell Wilder designed the classic KD for the treatment of epilepsy. The classic keto is a strict regime comprised of a 4:1 ratio, which means one part of carbs and proteins combined for four parts of fats. The use of KD for treating different diseases has increased over the past few decades. All the currently available versions are modified forms of classic KD. There are five types of KD published in the medical literature: (i) classic keto (ii) modified keto (iii) Medium-chain triglycerides oil (iv) Low glycemic index treatment (v) Modified Atkins diet. The macronutrient ratio is the major difference between these diets. In a nutshell, KD is a VLCD that relies on a moderate amount of proteins, high fat, and low carbohydrates that provide approximately 5–10% of calories from carbohydrates, 20–25% of calories from proteins, and 65–80% of calories from fats (28). KD includes fasting, proper hydration, physical activity, and intake of electrolytes and nutritional supplements (29).

The KD works by bringing certain metabolic changes to the body. Glucose is the body’s primary energy source. Carbohydrate deprivation resulting from KD causes a metabolic shift toward gluconeogenesis and ketogenesis. The preliminary shortage is managed by endogenous production of glucose from glycerol, glutamine, alanine, and lactic acid (gluconeogenesis). To keep up with the needs of the body, ketone bodies come into play and serve as an alternate energy source for the body (ketogenesis). At this stage due to low blood glucose feedback, secretion of insulin is also low, which further reduces the stimulus for fat and glucose storage. This ketotic state remains active until the body’s carbohydrates needs are fulfilled (30).

### Effectiveness of Ketogenic Diet

Literature is consistent with these findings that KD is an effective intervention for improving quality of life, seizure severity, and seizure frequency in epileptic patients (13, 31). KD is known for its neuroprotective action in various neurological illnesses like Alzheimer’s disease, amyotrophic lateral sclerosis, Parkinson’s disease, ischemic brain injury, traumatic brain injury, depression, autism, and narcolepsy (32). In the modern era, KD is recognized as a weight loss intervention but studies suggest mixed findings. A study compared the weight loss, appetite, and hunger responses of obese men who were fed a medium carbohydrate (35%) non-ketogenic diet (MCNKD) and low carbohydrates (4%) ketogenic diet (LCKD) in a crossover manner. After 4 weeks period, significantly greater weight loss and lower *ad libitum* energy intake were observed in the LCKD group because of reduced hunger (33).

A meta-analysis concluded that KD contributes to greater long-term weight loss than LFD (21). Another study compared the impact of KD and hypocaloric diet (HCD) on metabolic parameters in obese subjects. Fifty-eight subjects followed either of the two diets for 6 months. Greater differences in fat mass, weight, waist circumference, and fasting insulin were observed in the KD group as compared to the HCD group and only KD group showed significantly increased high molecular weight (HMW) adiponectin (34). The mechanism behind successful weight loss by KD is still a scientific debate. However, certain mechanisms have been hypothesized including appetite reduction due to the action of appetite-regulating hormones, fulfilling the effect of proteins, or appetite suppressing effect of ketone bodies (33, 35–37). Weight loss can also be due to the increase in lipolysis, reduction in lipogenesis, and ease in utilizing fats due to the increased metabolic efficiency as indicated by a reduction in the respiratory quotient at rest (38–42).

In a non-randomized controlled trial, type 2 diabetes mellitus patients received either an intervention diet (KD) or served as controls. The KD group lost 10–15% of body weight, had a reduction in inflammatory markers like hsCRP, decreased WBCs, and increased TGs, HDL-c, and LDL-c (43). A recent review summarized that despite the efficacy of KD for rapid weight reduction and improved HbA1c values, KD raise LDL-c and had no superiority over other diets in terms of safety, effectivity, and sustainability (44).

In a prospective study, KD promoted negative changes in lipoprotein sub-fractions. After 6 months, KD contributed to increasing the small LDL-c and decreasing the small HDL-c, thus increasing atherogenic risk in patients (45). In another study, 12 months of KD treatment was found to be associated with decreased carotid distensibility and increased LDL-c, TC:LDL-c, and LDL-c:HDL-c ratios. No significant changes were observed in hsCRP and BMI (46). Khodabakhshi et al. (47) evaluated the effect of KD on physical activity (PA), quality of life (QOL), and biomarkers in 80 metastatic breast cancer patients. In the 12-week trial, subjects were randomly allocated to either control or KD group. No significant differences in PA and QOL scores between the groups were reported. However, the KD group showed decreases in ALP and lactate levels.

### Health Consequences

Short-term minor side effects of KD are quite common, that include vomiting, nausea, gastrointestinal discomfort, fatigue, dizziness, feeling faint, decreased energy, and heartbeat alterations (48). KD initiation mostly results in hypoglycemia and lethargy (49). KD should be initiated with caution in combination with other treatments. A case report showed that the use of Valproate along with KD resulted in the development of hepatic dysfunction in a patient. The hepatotoxic effect was completely reversible as discontinuation of Valproate normalized the liver enzymes (50).

Ketogenic diet may negatively impact the lipid profile. A case report showed that following strict KD for 30–40 days, resulted in a rapid increase in LDL-c and TC. Fasting lipid profile showed HDL-c of 59 mg/dL, LDL-c of 199 mg/dL, TC of 283 mg/dL, and TG of 124 mg/dL. After discontinuation of KD and the use of statins for 4 weeks, there was a significant improvement in LDL-c (106 mg/dL) and TC (190 mg/dL). Furthermore, the patient maintained the optimal LDL-c levels after the discontinuation of statin therapy (51).

A recent case report demonstrated KD induced severe hyperlipidemia in an overweight 41 year old male. The patient had normal baseline values of lipid panel, i.e., LDL-c 99 mg/dL, HDL-c 49 mg/dL, TC 171 mg/dL, and TG 145 mg/dL. Following KD for 7 months resulted in severe hyperlipidemia as indicated by lab values, i.e., LDL-c 393 mg/dL, VLDL-c 41.5 mg/dL, HDL-c 54.4 mg/dL, TC 488.7 mg/dL, and TG 207.5 md/dL. Increasing the carbohydrates intake for 2 weeks, lipid panel showed remarkable improvement: LDL-c 279.0 mg/dL, VLDL-c 42.26 mg/dL, HDL-c 49.7 mg/dL, TC 371.2 mg/dL, and TG 211.3 mg/dL (52).

A retrospective cohort study showed that those on KD therapy had low normal bone mineral density, 8.8% of study subjects got kidney stones and 8.8% got a fracture during treatment (53). A newly recognized complication of KD is hypercalcemia. A series of case studies described the development of acute hypercalcemia about 2.1 years after initiating KD. Out of 14 patients, 13 had low levels of 1, 25-dihydroxyvitamin D, while all had low parathyroid hormone levels. Moreover, low alkaline phosphate (ALP) levels were noted in all subjects except the two oldest, while seven had impaired renal function (54).

## Paleolithic Diet (PD)

The PD also referred to as the Stone Age, caveman, or hunter-gatherer diet was initially introduced in 1985 by Eaton and Konner, and published by Dr. Loren Cordain in 2010 (55). It is marketed with the claims to improve health and cure diseases like obesity, cardiovascular disease, diabetes, cancer, and osteoporosis. Proponents of this dietary pattern believe that the modern diet (mainly processed foods, dairy products, grains, and legumes) is the cause of modern diseases and the obesity epidemic. Moreover, humans have evolved before agricultural development while the human diet has revolutionized more rapidly than our genetics; thus Paleolithic foods are more suited to our genetic makeup than the current modern diet (55, 56). Apart from this theory, anthropological research provides evidence that Paleolithic people used to eat a varied diet comprising of plants, grains, legumes, and game meats (57, 58).

Cordain’s PD has a basic set of rules, i.e., there is no restriction on the consumption of lean meats, fruits, and non-starchy vegetables while dairy products, legumes, cereals, and processed foods are strictly restricted (Table 2). There is little to no focus on portions, and calories. There are three adherence levels to the PD: entry-level, maintenance level, and maximal weight loss level (Table 3). One has a choice not to advance to the next level if satisfied with the results of this level (55).

**TABLE 2:** Foods in the Paleolithic diet.

Food groups	Foods allowed/restricted	Reference
Lean meat	About half of daily calories from lean animal foods are encouraged	(55)
Eggs	6–12 per week	
Fruits	All fruits are allowed Obese should be mindful of calories from high-sugar fruits	
Vegetables	All non-starchy vegetables are allowed	
Drinks and beverages	Mainly water Sugary beverages should be avoided Limited consumption of alcoholic beverages, i.e., two 4-oz servings of wine, 12-oz serving of beer or one 4-oz serving of spirits daily No tea or coffee	
Fats, oils and nuts	Unsaturated fats are allowed in moderation 4 Tbsp of oils per day 4 oz of nuts per day	
Vitamin and mineral supplements	Can be taken as per need	

**TABLE 3:** Levels of Paleolithic diet.

Levels	Description	Reference
Level 1: Entry level	3 open meals*/week Addition of some transitional foods** for the sake of improving compliance	(8)
Level 2: Maintenance level	2 open meals/week No transitional foods allowed	
Level 3: Maximal weight loss level	1 open meal/week	

*Open meal; flexible meals including foods from not avoid list, intended to improve the adherence to diet **transitional foods; food items that don’t meet Paleo rules.

### Effectiveness of Paleolithic Diet

Metabolic syndrome and insulin resistance are the prime focused areas in most of the literature related to PD. It does provide benefits but only to specific groups, i.e., eliminating dairy products can help people with digestive disorders. ‘Liberal consumption of fruits and vegetables can have a preventive effect for inflammatory bowel diseases (IBD). At the same time, this diet being high in meat increases the risk of IBD (59).

Paleolithic diet is powerful at advancing weight reduction for the time being, even at the point when the weight reduction is unintentional (60–62). Initially, weight loss is due to the loss of water weight as this diet is low in carbohydrates. Previous studies suggest that the study participants lost 4–6% of total body weight within 10–12 weeks (63, 64). Most of the studies are based on short-term interventions and there is only one study that followed the subjects for over 2 years. In a randomized trial, 70 post-menopausal obese women were divided into the *ad libitum* PD group or Nordic Nutrition Recommendations (NNR) diet group. After 24 months, the reductions in waist circumference, fat mass, and weight were observed in both groups irrespective of the dietary regimen followed (65).

Most of the studies reported the TC reduction properties of this diet while there are mixed results for HDL-c (61, 62, 64, 66). A study was conducted to evaluate the physiological and metabolic impacts of PD in healthy adults. After 10 days of intervention, reduction in TC, LDL-c, TG, and mean arterial pressure were observed (66). In another trial, participants were randomized to PD and reference diet groups. After 2 weeks of intervention, there were greater reductions in TC, TG, and diastolic blood pressure in the PD group (61).

In another study, healthy subjects followed this dietary intervention for 10 weeks, which resulted in increased LDL-c, TC, TC:HDL-c, along with a decline in HDL-c values (64). No significant changes in fasting blood glucose were seen in most studies (65, 66). While, some studies were short-term, where HbA1c was not measured as per protocol (67). Modest reduction, i.e., 3–4 mmHg in systolic or diastolic blood pressure was reported in most studies (60, 61, 66, 67). No significant change in inflammatory markers (CRP) was reported (61, 67).

### Health Consequences

The PD not only requires a big budget but is also very challenging to follow as compared to other diets (68, 69). Despite weight reduction and some favorable impact on cardiometabolic profile, this diet can have long-term consequences. Some studies suggest that this diet is not nutritionally balanced as it discourages certain food groups like whole grains, legumes, and dairy products. The micronutrient deficiencies can have long-term adverse outcomes. Those who follow PD have inadequate calcium intake. A study was conducted to check the nutritional adequacy of this diet. In addition to a low intake of carbs, fats, and total calories that could have promoted weight loss, this diet provided about 50% less calcium than the daily requirement (60).

Decreased HDL-c has also been observed among healthy adults and those with comorbidities. In a study comprising 28 type 2 diabetic patients, 14 followed the PD and 10 followed the American Diabetes Association (ADA) guidelines. Results showed that there was a significant reduction in HDL-c in the PD group (62). In another study, healthy subjects followed this dietary intervention for 10 weeks, which resulted in increased LDL-c, TC, TC:HDL-c, along with a decline in HDL-c values (64). More randomized trials need to be done to highlight the consequences of such diets that eliminate one or more food groups. PD is powerful at advancing weight reduction for the time being but its efficacy in cardiovascular events is not well established as limited long-term data is available.

## Mediterranean Diet (MD)

The concept of the MD emerged in the 1950s by Dr. Ancel Keys. In one of the first research that related diet and heart health, it was revealed that CVDs associated mortality rates are different in Westerns and Europeans. Lower mortality rates were observed in Europeans, even though they typically consume a moderately high-fat diet (70). Their dietary pattern can be linked to lower mortality and incidence of CVDs (71).

In 1975, Ancel Keys described this diet in his book as a complex of dietary choices followed by those living in Mediterranean regions. Whole grains, legumes, fruits, vegetables, olive oil, fish, and nuts are key components of this diet with a moderate allowance of alcohol, dairy products, and meat [Keys, (72)]. Traditionally, this diet derives its most calories from fish and plant-based foods. Fats account for 30% of calories which are mostly polyunsaturated fatty acid (PUFA) and monounsaturated fatty acids (MUFA), while carbohydrates provide 50–55% of calories from low glycemic index carbohydrates and proteins provide 15–20% of calories (73).

### Effectiveness of Mediterranean Diet

Mediterranean diet is the most extensively studied diet to date. In a previous review, it has been summarized that MD is nutritionally adequate for the general public and may have the potential of preventing micronutrient deficiencies (74). Research shows that it has preventive and therapeutic potential for many chronic diseases like non-alcoholic fatty live disease (NAFLD), CVDs, metabolic syndrome, and certain cancers like colorectal and breast cancer (75). In a 2-year trial, weight loss by LFD, AD, and MD was compared and results showed that the AD group had the highest mean weight loss, i.e., – 4.7 ± 6.5 kg, while the MD group stood second with a mean weight loss of – 4.4 ± 6 kg and LFD group lost –2.9 ± 4.2 kg. Following changes were recorded in the MD group: increased molecular adiponectin reduced serum leptin, and CRP levels (76).

In another controlled trial, 259 subjects were randomly allocated to American Diabetic Association (ADA) diet, traditional MD, or low carbohydrate Mediterranean diet (LCM) group. After 12 months, the LCM group had the highest weight reduction, increased HDL-c, improved LDL-c, TG and HbA1c (77). Another study described the effectiveness of MD in the primary prevention of CVDs [Estruch et al. (78)]. A study investigated the protective effect of MD against cancer and found that greater compliance with MD patterns reduces the risk of non-tobacco linked cancers in both men and women (79).

Most MD studies are short-duration studies, only a few studies focused on the long-term impacts of following MD. In a study, non-diabetic elderly subjects (*n* = 3,541) at higher risk of CVD were randomized to three intervention groups: control diet, MD with nuts or MD with extra virgin olive oil. New cases of diabetes were recorded after regular intervals (median follow-up duration = 4.1 years) and results showed that MD with extra virgin olive oil was associated with a reduced risk of diabetes (80). In another 5 years clinical trial, subjects (*n* = 7,447) who were type 2 diabetics or those with risk factors of CVDs, were randomized to three intervention groups: control diet, MD with nuts, or MD with extra virgin olive oil. After the specified period, there was no significant weight reduction in all the groups, while the MD group had a significant reduction in central obesity [Estruch et al. (81)].

### Health Consequences

No evidence of adverse effects associated with MD is available in the literature. Rather, MD has preventive and therapeutic potential for many chronic diseases. It is highly suitable for the general public for the prevention of micronutrient deficiencies and specifically for those patients who are more health-conscious than just weight loss oriented.

## Vegetarian Diet (VD)

**FIGURE 2:**
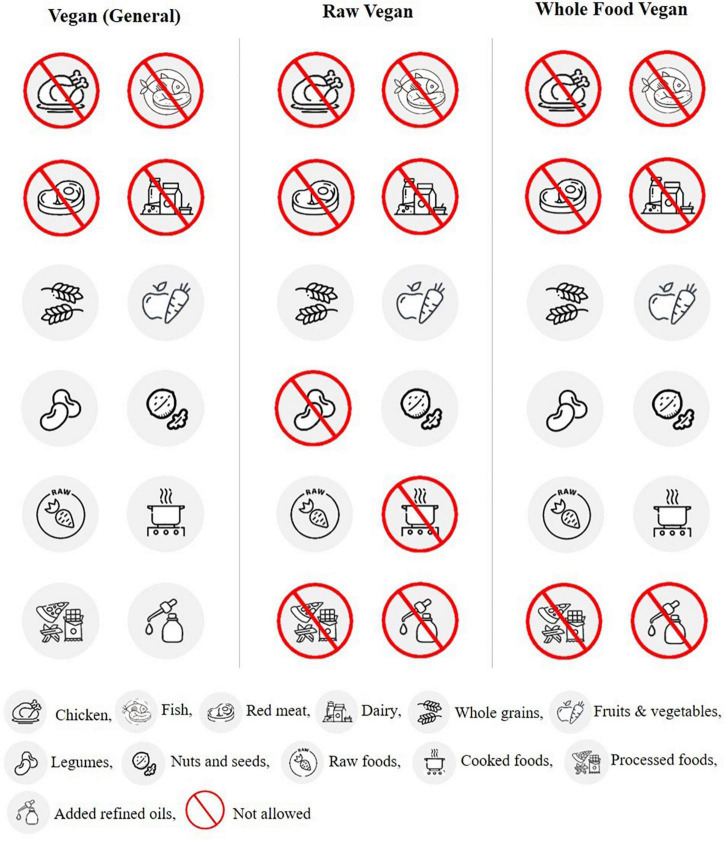
Foods allowed and restricted in vegan diets.

The VD is a dietary pattern characterized by no consumption of meat and meat products, seafood, poultry, and sometimes other animal products like eggs, animal milk, and honey. Some studies have linked meat intake with an increased risk of chronic diseases, while others indicate a positive association between low meat intake and life expectancy (82, 83). VD are of four main types: (i) a lacto-ovo-vegetarian does not consume any meat product but consumes eggs and dairy products (ii) a lactovegetarian does take dairy products but does not consume eggs and meat products (iii) an ovo-vegetarian does not eat meat products and dairy products and are free to consume eggs (iv) a vegan does not consume any animal products, including meat, eggs, dairy products, and honey (84). Vegan diets include different subtypes: raw vegan, vegan (general), and whole-food vegan (Figure 2). Each subtype has its own set of foods allowed and restricted with one thing in common, i.e., meat products restriction. This dietary pattern is gaining much popularity in the general population, especially in the Western world (85). There are various reasons for adopting this dietary profile, including religious beliefs, ethical motivation, cultural aspects, and health considerations (85, 86).

### Effectiveness of Vegetarian Diet

Several epidemiological studies reported a lower cardiometabolic risk in the vegan population. A study concluded that non-vegetarians have a higher type 2 diabetes prevalence (7.6%) than vegetarians (2.9%). While, the prevalence rate also varies with the type of VD, i.e., 3.2% in lacto-ovo vegetarians, 4.8% in pesco-vegetarians and 6.1% in semi-vegetarians. This can be explained by the low-glycemic-response associated with these diets as vegetarian diets typically include foods that have a low glycemic index such as beans, legumes, nuts, some fruits and vegetables (87). Glycemic control via a VD is quite controversial, as these are high carbohydrate diets. Some studies have shown that vegetarians also have increased life expectancy (82). Generally, vegetarians are more health-conscious and have lower BMI than the general population (88). The Seventh Day Adventist study showed a lower mean BMI, i.e., 23.6 kg/m^2^ in the vegan population (89). In a 5-year prospective study, 22,000 subjects having different dietary patterns were checked for their weight gain during this period. Vegans had the lowest weight gain as compared to meat-eaters and fish eaters (90).

Red meat and poultry intake were most strongly linked to increased risk of esophageal adenocarcinoma and gastric cardia or non-cardia adenocarcinoma, respectively (91). On the other hand, lower rates of heart diseases and cancers have been observed in vegetarians in comparison with those following other dietary patterns (92, 93). A better cardiometabolic risk profile is generally present in vegetarians, i.e., lower BMI, TC, and LDL-c [Chen et al. (94); De Biase et al. (95)]. A cross-sectional study investigated the lipid profile of fish-eaters, meat-eaters, and vegetarians. Not only the vegans have a lower BMI but also favorable serum lipid levels: lower LDL-c, TC, and apolipoproteins (96).

In a study, out of 26,346 participants, 1,079 cases of prostate cancer were identified and results showed the protective effect of vegan diets against prostate cancer in the white population (97). This protective effect against prostate cancer may be due to the higher fiber intake. Some other studies are either short-term or have a very small sample size, showing mixed findings related to colorectal cancer and breast cancer. In a study, 2,304 patients from 10 European countries were assessed for their dietary intake to find the impact of diet on the risk of cancers. Not poultry but red meat intake was found to be associated with an increased risk of esophageal cancer and upper aerodigestive tract (UADT) cancer. Furthermore, vegetable and fruit intake are significantly associated with a reduced risk of UADT cancer (98).

Butler et al. (99) demonstrated that the higher the intake of vegetable-fruit -soy dietary pattern, the lower the breast cancer hazard ratio among postmenopausal women. Another study showed a significant association between consumption of vegetables and risk of esophageal adenocarcinoma. Elimination of potentially harmful dietary components like animal protein, saturated fats, and cholesterol can be the reason for these benefits. These benefits can also be due to the addition of dietary fiber, phytochemicals, and antioxidants rich in beneficial dietary components like whole grains, legumes, nuts, fruits, and vegetables (91).

### Health Consequences

This diet is associated with fluctuations in micronutrients intake because of the day-to-day variation in the menu. Depending upon the type of VD, vegetarians are potentially at increased risk of micronutrient deficiencies such as calcium, zinc, iron, vitamin E, vitamin B12, essential fatty acids, docosahexaenoic acid (DHA), and eicosapentaenoic acid (EPA) (100). A study reported that half of the vegan participants were micronutrient deficient as compared to omnivores (101). Vegetarians have lower serum vitamin B12 levels as plant sources are deficient in this vitamin (101, 102). As VD is generally low in calcium due to the suboptimal intake of dairy, vegetarians are at greater risk of bone fractures due to the lower bone mineral density (103).

Thus supplementation of certain vitamins like vitamin B12 and vitamin D is needed to avoid these deficiencies among vegetarians (84). Vitamin B12 supplements are especially important for vegan pregnant and lactating mothers as a preventive therapy for deficiency in their babies (104). VD can be nutritionally adequate, so it may be helpful in chronic disease prevention and treatment. Benefits and harms depend upon the dietary choices so the individualized plan fulfilling the micronutrient requirements must be carefully developed by a professional.

## Intermittent Fasting (IF)

The IF is gaining much popularity and is widely adopted as an effective weight loss intervention. Contrary to the conventional weight loss programs that are based on calorie restriction, IF is more about scheduled eating. Some of the key features of IF are abstinence from food for a certain period, followed by a period of normal eating. There are various versions of IF but the most popular of these are alternate day fasting (ADF), 5:2 diet or periodic fasting (PF), and time-restricted feeding (TRF). The frequency and duration of fast cycles may differ among all types (Table 4).

**TABLE 4:** Types of intermittent fasting.

Types	Description	Fasting definition	Normal eating	Reference
Alternate day fasting	Fasting alternated with a day of normal eating	0–25% of TCN*	*ad libitum*	(105)
5:2 diet or periodic fasting	Fasting for 2 days with normal eating for 5 days	0–25% of TCN*	*ad libitum*	
Time-restricted feeding	Normal eating within a window of < 8 h per day	–	*ad libitum*	

*TCN, total caloric needs.

### Effectiveness of Intermittent Fasting

The alternate-day fasting (ADF) approach has been tested for its metabolic effects. In a study, healthy young men (*n* = 8) were subjected to ADF for 20 h/day for 15 days. After the specified study period, weight remained unchanged (86.4 ± 2.3 kg) while the increase in glucose uptake, i.e., 7.3 ± 0.3 mg/kg/min that was previously 6.3 ± 0.6 mg/kg/min, and prominent increase in lipolysis of adipose tissues were observed (106). Another study showed that when non-obese subjects (8 women and 8 men) fasted for 22 days on alternate days, they lost 4 ± 1% of their initial fat mass and 2.5 ± 0.5% of their initial body weight. However, a decrease in fasting insulin and non-significant change in glucose and ghrelin were also reported (107).

A randomized crossover trial was conducted to evaluate the fasting-induced acute changes in biomarkers. Healthy volunteers (*n* = 30) were randomized into two groups: (i) normal eating for 28 ± 4 h then water-only fasting for 28 ± 4 h (ii) 28 ± 4 h of water-only fasting then 28 ± 4 h of normal eating. Blood samples were drawn and analyzed at baseline, day 1 and day 2. Laboratory findings suggested that the fasting intervention acutely increased hemoglobin, hematocrit, red blood cell count, human growth hormone, and HDL-c; on the other hand, decreased body weight, bicarbonates, and TGs, as compared to the normal eating day. Moreover, cholesterol and human growth hormone returned to baseline after 48 h (108).

Night-time fasting (NTF) has been linked to lower energy intake, consequently resulting in weight loss. In a study, twenty-nine healthy young men were subjected to 9 h of NTF for 2 weeks, then 1 week washout period followed by 2 weeks of controlled conditions. Results showed that the participants had less total calorie intake in the NTF phase as compared to controlled conditions. Significant differences in weight change were also reported, i.e., – 0.4 kg for NTF and + 0.6 kg for control (109).

In a randomized trial of 3 months, young overweight premenopausal women (*n* = 107) were randomly assigned to two groups: two consecutive days of fasting (25% energy restriction)/week or fasting for all days of the week. Both interventions were found to be equally good at a weight and showed improvement in risk markers of CVDs, cancer, and diabetes for example reduction in leptin, leptin to adiponectin ratio, inflammatory markers, fasting insulin, insulin resistance, blood pressure, and lipids (110).

Fasting also impacts the appetite by influencing the appetite-regulating hormones (110, 111). A previous systematic review summarized that IF may have the potential to provide metabolic benefits in terms of improving insulin resistance, thus providing better glycemic control as IF showed a significant decline in fasting glucose levels as compared to controls. Moreover, IF was associated with a decline in BMI, fat mass, and leptin while an increase in adiponectin (112). Headland et al. (113) evaluated the effectiveness of intermittent energy restriction (IER) in improving weight and biological markers in long-term studies. Irrespective of duration, IER was associated with weight loss. However, IER was not found to be superior to continuous energy restriction (CER) in terms of weight loss, blood lipids, glucose, and insulin levels.

### Health Consequences

Some short-term studies highlighted the potential harms posed by IF among normal-weight subjects. IF induces lipolysis, resulting in increased free fatty acids (FFA). So whether it be ADF, periodic fasting, or else, a prolonged course of fasting can lead to large fluctuations in FFA in normal-weight individuals. A study showed that these fluctuations were three times greater than those typically seen after an overnight fast. Furthermore, it induced reductions in insulin sensitivity and acute glucose-simulated insulin response (114). Despite the effectiveness of IF in weight loss as indicated by several studies, the current evidence is non-conclusive. The prime focus of available literature is weight loss but little is known about its sustainability and long-term health effects. More long-term trials should be conducted to draw a clear conclusion.

## Detox Diets (DD)

The popularity of detoxification dates back to Greek, Roman, Indian, and Native American cultures. Many effective approaches that are still used for the removal of toxins include fasting, saunas, herbs, rebounding, dry brush, water, rest, exercise, and meditation (115). However, detoxification or DD are interventional diets specifically designed for toxins elimination, health promotion, and weight management. These short-term dietary interventions involve multiple approaches, including total calorie restriction, dietary modification, or juice fasts, and often involve the use of additional minerals, vitamins, diuretics, laxatives, or cleansing foods. Some commercial DDs have been listed in Table 5. These are most commonly prescribed by naturopathic doctors to prevent or treat a number of conditions like gastrointestinal disorders, inflammation, autoimmune disorders, chronic fatigue syndrome, fibromyalgia, and weight loss (116).

**TABLE 5:** Commercial detox diets.

Diet type	Duration	Foods allowed	Proposed claims	References
Liver cleansing diet	8 weeks	Plant-based, dairy-free, low fat, high fiber, unprocessed foods are allowed. Epsom salt and liver tonics are also consumed.	Improved energy levels and liver function Toxins removal Improved immune response Efficient metabolism of fats and better weight control	(117)
Lemon detox diet/Master cleanser	10 days	A liquid only diet based on purified water, lemon juice, tree syrup and cayenne pepper. A mild laxative herbal tea and sea salt water is also incorporated.	Toxins removal Shiny hair, glowing skin and strong nails Weight loss	(118)
The clean cleanse	21 days	Breakfast and dinner comprise probiotic capsules, cleanse supplements and cleanse shakes. A solid meal in lunch while avoiding gluten, dairy, corn, soy, pork, beef, refined sugars, some fruits and vegetables.	Toxins removal Improved energy, digestion, sleep and mental health Reduction in joint pains, headaches, constipation and bloating	(119)
Martha’s vineyard detox diet	21 days	Herbal teas, vegetable soups and juices, specially formulated tablets, powders and digestive enzymes are on the menu.	Weight loss up to 9.5 kg Toxins removal Improved energy levels	(120)
Weekend wonder detox	48 h	Protein-rich meals salads, detox-promoting super foods and beverages. Healthy lifestyle, spa treatments and herbal remedies.	Toxins removal Improved organs’ function Strengthen body Enhance beauty	(121)
Fat flush	2 weeks	Large meals are replaced with dilute cranberries, hot water with lemon, pre-prepared cocktails, supplements and small meals	Toxins removal Reduced stress Weight loss Improved liver function	(122)
Blue print cleanse	3 days	Consumption of six pre-prepared vegetable and fruit juices is allowed per day.	Toxins removal	(123)
The Hubbard purification rundown	Several weeks	Niacin doses along with sustained consumption of vitamin-A, B, C, D, and E. Daily exercise with balanced meals. Restriction of alcohol and drugs. Sitting in a sauna for ≤ 5 h each day.	Toxins removal from fat stores Improved memory and intelligence quotient Better blood pressure and cholesterol levels	(124)

The DDs have not been extensively investigated; however, the handful of available studies have methodological limitations like sampling bias, small sample sizes, relying on self-reporting, and absence of control groups. Despite the emerging popularity, these diets fail to identify the mechanisms of eliminating toxins or even the specific toxins removed by a particular diet. Detox approaches defy the general principles of human physiology as the liver and kidneys are quite efficient in removing both exogenous and endogenous toxins from our body, along with extra-renal excretion of toxins in sebum and sweat (125).

### Effectiveness of Detox Diets

Currently, there is no clinical evidence confirming or negating the effectiveness of commercially available detox regimes for losing weight. Because of its emerging popularity, this area needs attention. So, in the absence of scientific evidence, results can be extrapolated from other closely related studies. It is known that the success rate of dieting, in general, is only 20% (126). This may be possible because humans and animals have natural mechanisms to counter the weight loss as starvation can have negative health consequences like reduced fertility and even death. Calorie restriction alters the neuropeptides’ expression in the hypothalamus; which reduces metabolic rate and stimulates appetite, resulting in a weight loss plateau (127).

Furthermore, studies in mice have shown binge eating followed by a period of energy restriction, though this phenomenon is not established in humans yet (128). A study conducted by Mazurak et al. (129) showed that fasting raised cortisol levels in young healthy women. Another study reported an increase in stress hormone levels in females due to the restricted intake of 1,200 kcal/day (130). There is considerable evidence that stress stimulates appetite, thus promoting weight gain via elevations of cortisol (131).

Many of the DD are liquid-based, low-calorie, and nutrient-poor. For example, a part of BluePrint Cleanse, Excavation Cleanse, provides only 19 g protein and 860 kcal/day which is far below the actual requirement. Food and Agriculture Organization (FAO) recommends a minimum of 0.83 g/kg body weight of high-quality protein and 1,680 kcal/day for an adult (132, 133). Based on the previous work, DD may induce stress, raise cortisol levels and increase appetite, resulting in difficulty in losing weight, followed by binge eating and weight gain (128–130).

It is quite alarming that the components of detox products may not be according to the labels as there is no regulatory authority that approves such products. A case was reported in Spain that a 50 year old man with no history of relevant medical illness, presented with diffuse abdominal pain, lethargy, profuse diarrhea, and vomiting after ingesting Epsom salt during a liver cleansing diet. That person died within 72 h from the onset of symptoms. Forensic and clinical investigations concluded that instead of magnesium sulfate heptahydrate, the supplier had mistakenly added hydrated manganese sulfate resulted in manganese intoxication (134).

Energy-restricted DDs are capable of short-term weight loss. But still, there is a high likelihood of health risks from detox products because of their nutritional inadequacy. As no convincing evidence exists in this domain so such diets and products need to be discouraged by health professionals and must be subjected to regulatory review and monitoring.

## Conclusion

Fad diets facilitate fast and easy weight loss, improve appearance, and do not require a longer time to achieve the results. These diets are effective in improving health to some extent. However, compliance is always a significant concern because of the unrealistic combinations and nutritional inadequacy due to the complete elimination of one or more essential food groups. Despite the rapid weight reduction, there are some concerns for those with comorbidities. All these diets have not been extensively studied while those studies that have been mentioned in the literature have high dropout rates and are sometimes non-conclusive. More randomized controlled trials of prolonged duration need to be done to establish the safety of FDs for the public and to make people aware of the possible consequences of long-term adherence to such dietary patterns.

## Author Contributions

AT and AR: conceptualization. AT: writing – original draft preparation. AT, AN, RR, AR, RA, CS, and CM: writing – review and editing. AR and RA: supervision. All authors have read and agreed to the published version of the manuscript.

## Conflict of Interest

The authors declare that the research was conducted in the absence of any commercial or financial relationships that could be construed as a potential conflict of interest.

## Publisher’s Note

All claims expressed in this article are solely those of the authors and do not necessarily represent those of their affiliated organizations, or those of the publisher, the editors and the reviewers. Any product that may be evaluated in this article, or claim that may be made by its manufacturer, is not guaranteed or endorsed by the publisher.
